# At the vanguard: Leaders’ perspectives on establishing healthcare system infection prevention programs

**DOI:** 10.1017/ash.2022.334

**Published:** 2022-12-09

**Authors:** Michael P. Stevens, Sharon B. Wright, Keith S. Kaye, Jerry M. Zuckerman, Catherine L. Passaretti, Richard A. Martinello, Hilary M. Babcock, Michael B. Edmond, Graham M. Snyder

**Affiliations:** 1 Division of Infectious Diseases, West Virginia University School of Medicine, Morgantown, West Virginia; 2 Division of Infectious Diseases, Beth Israel Deaconess Medical Center, Boston, Massachusetts; 3 Division of Infectious Diseases, Rutgers Robert Wood Johnson Medical School, New Brunswick, New Jersey; 4 Department of Internal Medicine, Hackensack Meridian School of Medicine, Nutley, New Jersey; 5 Atrium Health, Charlotte, North Carolina; 6 Departments of Internal Medicine and Pediatrics, Division of Infectious Diseases, Yale School of Medicine, New Haven, Connecticut; 7 Division of Infectious Diseases, Washington University School of Medicine, St. Louis, Missouri; 8 Division of Infectious Diseases, University of Pittsburgh Medical Center, Pittsburgh, Pennsylvania

## Abstract

Hospitals are increasingly consolidating into health systems. Some systems have appointed healthcare epidemiologists to lead system-level infection prevention programs. Ideal program infrastructure and support resources have not been described. We informally surveyed 7 healthcare epidemiologists with recent experience building and leading system-level infection prevention programs. Key facilitators and barriers for program structure and implementation are described.

The United States has a shortage of trained healthcare epidemiologists. The field of infectious diseases overall has had trouble recruiting physician trainees in recent years, with only 82% of fellowship positions being filled in the 2021 match.^1^ Healthcare epidemiology as a subspecialty of infectious diseases does not have an accredited fellowship program, and only a few nonaccredited, dedicated fellowship programs exist. No estimate of how many formally trained healthcare epidemiologists currently work in the United States has been published.

The COVID-19 pandemic has highlighted the critical importance of the fields of healthcare epidemiology and infection prevention. Coinciding with this heightened awareness and appreciation for infection prevention specialists were Herculean demands that have contributed to burnout and infection prevention workforce erosion.^2^

Hospitals are increasingly consolidating into health systems.^3^ With this expansion has come a need for system-level coordination of key programs. Some health systems have appointed system healthcare epidemiologists to oversee system-level infection prevention activities. Best practices for healthcare epidemiologist–led, health-system–wide approaches to the prevention of healthcare-associated infections (HAIs) are urgently needed. We interviewed 7 healthcare epidemiologists with recent experience leading and building health system-based infection prevention programs who self-identified in response to a query on a longstanding e-mail group of healthcare epidemiologists. These informal surveys were used to identify several key themes (Table 1).

**Table 1.** Key Facilitators and Barriers for System Infection Prevention Program Implementation and Function

Area	Key Elements
Facilitators	**Staff support** Inclusion of a system infection prevention director Ideally a 1.0 full-time equivalent (FTE) position Protected time for the system healthcare epidemiologist Ideally would have a minimum protected FTE of 75% Support needs will vary by system size and other factors Dedicated information technology specialist support This includes both programming and analyst support Dedicated administrative assistant support **Data and technology** Single, integrated electronic medical record (EMR) system Data automation Adoption of virtual meeting platforms Accelerated by COVID-19 pandemic Several survey respondents noted virtual meetings had supplanted in-person meetings almost entirely **Network characteristics** System exists in a limited geographic area Facilitates travel within the network Community building activities with infection preventionists within the system as well as regular meetings
Barriers	**Cohesion issues** “Us versus them” perception: System healthcare epidemiologist is primarily affiliated and spends most of their time at a single system facility Between academic and nonacademic facilities **Data and technology** Heterogeneity of electronic medical record systems Associated with difficulty extracting data and creating reports Increases difficulty with supporting individual facilities in real time with things like surveillance questions, outbreak investigation, etc **Network characteristics** Rapidly expanding healthcare systems Multistate systems Adds complexity with state-specific regulations Systems spread over a wide geographic area

The hospital epidemiologists who were interviewed had been in their system healthcare epidemiology leadership roles for an average of 5.4 years (range, 1–11). Protected time ranged from 50%–100% (mean, 79%). None of the interviewees had clinical responsibilities in an outpatient clinic, and inpatient service responsibilities ranged from 0 to 10 weeks per year (mean, 5.6). The number of inpatient facilities covered in each health system ranged from 5 to 40 (median, 13), and all were also responsible for outpatient facilities. Also, 4 hospital epidemiologists (57%) were responsible for systems spanning multiple states.

In addition to the key facilitators and barriers identified in Table 1, several other key themes emerged. Lack of data access was a challenge. Although most systems produce system-level HAI-outcome dashboards and report on hand hygiene compliance, none regularly produced comprehensive system-level process-measure dashboards. Most of the respondents reported through their system’s chief quality officer or chief medical officer and had limited direct authority over system personnel. Identifying local culture and engaging local stakeholders were considered critically important. Community building among infection prevention personnel was also considered vital. Many of the respondents also served as the primary healthcare epidemiologist for individual system facilities (often academic hospitals). This overlap could lead to concerns about bias by some system facilities, which could undermine influence and negatively impact team building. Many were working to create system-level infection prevention policies and noted that COVID-19 had facilitated this work. A few systems had adopted centralized HAI surveillance.

System healthcare epidemiologists added value in several ways: overseeing data quality; report standardization; establishing, implementing, and assuring compliance with best practices; sharing knowledge gained from HAI events and regulatory surveys; providing expertise with product value analysis; advocating for infection preventionist support locally and at the system level; and promoting research and innovation. Policy unification and data automation were identified as future opportunities. Many programs also planned on adopting centralized HAI surveillance. Additionally, a goal of multiple programs was reporting system-level process metrics.

Data on system-level infection prevention programs are limited. Barnes et al^4^ surveyed a group of corporate and system-level directors of infection prevention in 2016. Of the 32 respondents, only 37% had physician support funded for their corporate programs. The training background for the involved physicians and their roles within these programs was not described. Although the survey did not explore the potential advantages of physician engagement in system-level infection prevention programs, these researchers postulated that physician involvement would benefit programs in several ways. These benefits included influencing executives and physicians with practice change, assistance with analyzing data and assessing risk, helping to set program goals, and supporting individual facilities lacking direct physician support. The respondents noted varied program structure and support, but most had the support of a data analyst (1–4 FTEs). Most corporate infection prevention directors (75%) had some sort of direct authority over infection preventionists in individual facilities.^4^

Infection prevention and antimicrobial stewardship programs share similarities in strategies, infrastructure, and key metrics.^5^ Buckel et al^6^ published an analysis of 20 system-level antimicrobial stewardship programs. They identified 4 program models ranging from centrally led teams with formal system-level leadership, resources, and participation requirements to collaborative teams with no formal structure and voluntary participation. Barriers to implementing centralized stewardship programs included inadequate data infrastructure and staff funding as well as securing local facility participation.^6^ Inadequate data infrastructure and staff funding support were also noted as key barriers to system infection prevention program creation in our informal survey.

We hope that our experience will be of value to those considering system healthcare epidemiologist positions. Ultimately, research is needed to determine the impact of system coordination on HAI reduction and healthcare worker safety. Additionally, key resources and training standards should be delineated. System-level infection prevention programs have enormous potential to capitalize on the knowledge and expertise of the limited number of trained healthcare epidemiologists and to expand and transform HAI prevention.
